# Treatment of Recurrent Wide Neck Bifurcation Aneurysm With the Barrel Vascular Reconstruction Device

**DOI:** 10.3389/fneur.2019.01159

**Published:** 2019-11-05

**Authors:** Stanimir Sirakov, Adriana Panayotova, Alexander Sirakov, Karsimir Minkin, Kirstian Ninov, Radoslav Raychev

**Affiliations:** ^1^University Hospital St. Ivan Rilski, Sofia, Bulgaria; ^2^Department of Neurology, University of California, Los Angeles, Los Angeles, CA, United States

**Keywords:** stent-assisted coil embolization, brain aneurysm, wide neck aneurysm, subarachanoid hemorrhage, retreatment

## Abstract

We present a case of successful embolization of a recurrent wide neck bifurcation aneurysm with a Barrel vascular reconstruction device (VRD). The unique properties of this novel device allowed optimal aneurysm neck coverage during third consecutive re-treatment, ultimately resulting in complete aneurysm obliteration. The parent vessel anatomy and the neck morphology of the aneurysm, in combination with a presence of a large pre-existing coil mass, were ideal for Barrel stent placement. The expanded portion of the device conformed perfectly to the recanalized aneurysm neck, providing optimal support for additional coil embolization. This case illustrates the advantages of Barrel VRD for definitive embolization of large, recurrent, and previously coiled wide-neck bifurcation aneurysm as a reasonable alternative to other traditional treatment modalities, such as flow diversion or Y and X stenting.

## Introduction

Despite the recent introduction of multiple novel intra-saccular and flow-diversion devices and techniques, endovascular treatment of wide neck bifurcation intracranial aneurysms remains challenging, especially in setting of recurrence after prior coil embolization. We present a case report of complete obliteration of recurrent wide-necked aneurysm of the Anterior Communicating Artery, treated with the assistance of the Barrel VRD. This relatively new stent is designed specifically for optimal single-device neck protection during coil embolization, aimed to minimize the metal-to-wall ratio associated with other traditional techniques. Furthermore, its unique expanded design is particularly favorable in cases of previously treated recanalized aneurysms with large intrasaccular coil mass.

## Materials and Methods

### Patient Information

**Figure 1:**
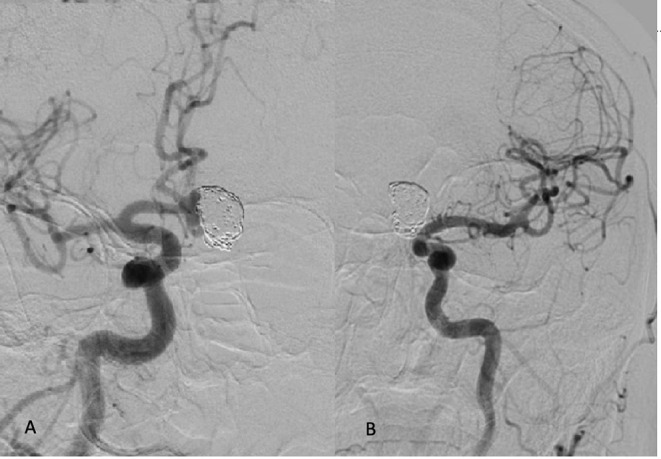
**(A)** Recanalization of the aneurysmal sac 6 months after the second coiling procedure seen from R ICA injections in oblique projection. Both ACAs arise from the dominant R A1 segment. **(B)** Left ICA injection demonstrates hypoplastic L A 1 segment.

A written informed consent was obtained from the patient for the publication of the present case report. A 56 years old patient was initially transferred to our facility with a ruptured large saccular aneurysm of the Anterior Communicating artery. The patient presented with severe lethargy, headache, nausea, vomiting, and confusion, compatible with Hunt and Hess grade 3 subarachnoid hemorrhage (SAH). CT scan confirmed presence of Fisher grade 3 SAH. CT angiography demonstrated a large saccular aneurysm of the Anterior Communicating artery, measuring 19 × 15 mm at the dome and 6.9 mm at the neck. Balloon assisted coiling was chosen as a first treatment option, using the Scepter C balloon (Microvention, USA) and several Target coils (Stryker Neurovascular, USA) resulting in satisfactory embolization of the aneurysm. The patient recovered well after rehabilitation. Follow up control digital subtraction angiography (DSA) demonstrated significant recanalization of the aneurysm, requiring second embolization. The aneurysm was treated with several additional Axium Prime coils (Medtronic, USA), again resulting in adequate embolization. However, the two following consecutives DSAs in 3 and 6 months after the most recent embolization demonstrated a recurrent recanalization at the base of the aneurysm (Figure 1). Given the wide-neck configuration and the location at the A1/A2 bifurcation, the Barrel VRD was chosen as the best strategy for additional stent-assisted coiling with a plan to position the expanded portion of the stent at the base of the aneurysm. Other strategies, such as Y stenting or flow diversion were also considered but felt to be much riskier due to high metal coverage of the arterial wall and high potential for inadequate flow diversion as both A2 were incorporated in the aneurysm base.

The patient was placed on dual antiplatelet therapy prior to the endovascular treatment with Aspirin and Clopidogrel 5 days before the planned procedure. Platelet inhibition was assessed by the P2Y12 test and was proven to be adequate.

### Endovascular Technique

**Figure 2:**
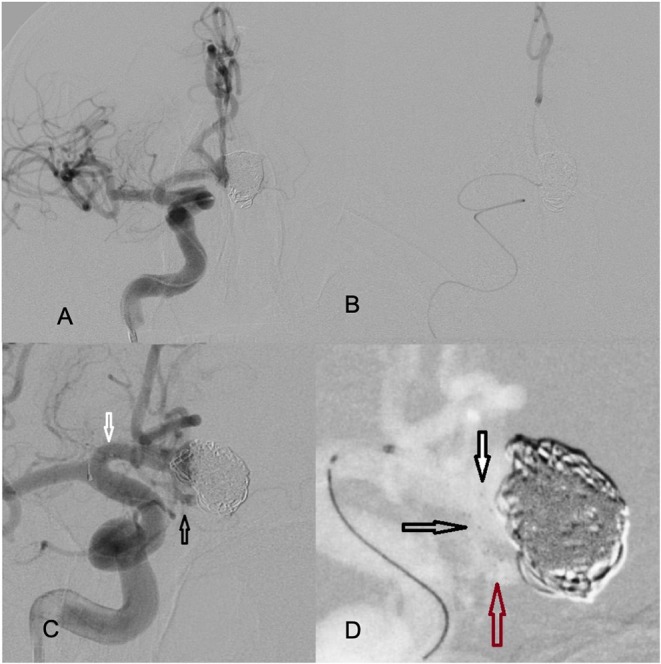
**(A)** The Rebar microcatheter extends from R ICA to the contralateral left A2 segment. **(B)** The positioning of the distal tip of the microcatheter in the L A2 is confirmed by selective injection. **(C)** The stent was deployed from the proximal left A2 segment (black arrows pointing at distal markers) to the proximal R A1 segment (white arrows pointing at the proximal markers). **(D)** Magnified view of the near-completely deployed device. The expanded portion of the device fully conforms to the neck (white and black arrows). The red arrow points to the distal end of the stent positioned at the proximal left A2 segment.

**Figure 3:**
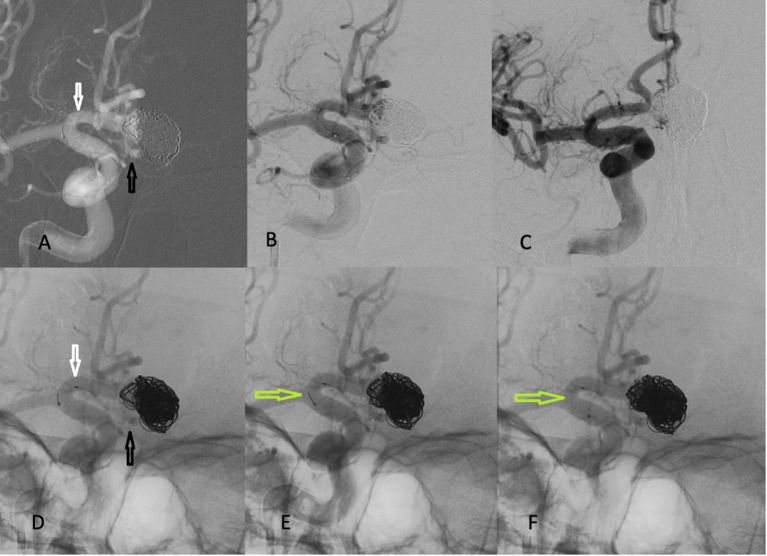
Embolization of the recanalized portion of the aneurysm with coils. The bottom images are un-subtracted for better visualization of the stent and coils. **(A,B,D)** The Barrel VRD is positioned from proximal R A segment (white arrow) to the proximal L A2 segment (black arrow). **(E,F)** The stent was detached (green arrows), once coiling was completed. **(C)** Final angiogram in working projection demonstrating complete obliteration of the aneurysm.

Under general anesthesia, the right common femoral artery was catheterized using the Seldinger technique. Using a 6 Fr. Chaperon guiding catheter (Microvention, USA), a Rebar microcatheter (EV3, USA) was navigated over a Silver Speed guidewire (EV3, USA) into the R A 1 segment. The guide catheter remained in the distal cervical ICA. The Barrel stent was deployed by positioning the bulged segment across the aneurysmal neck using its 12 radiopaque markers as a reference. The distal end of the device was positioned in the left A2 segment, and the proximal end of the device extended into the proximal R A1 segment (Figure 2). A second microcatheter (Echelon 10) was then delivered over the Silver Speed guidewire across the stent into the recanalized portion of the aneurysm. Several Axium Prime coils were deployed until full occlusion of the aneurysm was achieved (Figure 3).

## Results

The postoperative period was uneventful, and the patient was discharged home in stable condition without any neurological deficits. The patient remained on dual antiplatelet therapy for total of 6 months.

An early follow up DSA was obtained 3 months after the Barrel VRD-assisted coiling, showing complete obliteration of the aneurysm. The durability of the treatment was confirmed by subsequent angiograms at 6 and additional 9 months, totaling 15 months of angiographic follow up after the final embolization.

## Discussion

Endovascular treatment of wide-necked intracranial aneurysms (WNA) has always been a challenging task. Historically, all aneurysms with neck diameter exceeding 4 mm were considered difficult for primary coil embolization (1). Further studies have defined the fundus/neck ratio ≤ 2 as the main prognostic factor for technical challenges and potential risk of complications (2). The aspect ratio, defined as the aneurysm height/neck ratio, has also been identified as an independent predictor of treatment type, estimating that 89% of aneurysms with aspect ratios ≤ 1.2 require adjunctive devices for successful coil embolization (3). In our case, the aneurysm met all those criteria.

Y-stenting is one of the first techniques aimed to improve coil embolization success for WNA. Two stents are deployed in parallel (“kissing configuration”) or inside one another (“crossing” configuration) forming a Y shape and thus securing the parent vessels from coil protrusion (4, 5). Another option for treating WNAs is the double balloon remodeling (6). However, there are several disadvantages of these techniques, including placement of multiple microcatheters, distal ischemia during the balloon occlusion, high metal-to-wall ratio and potentially suboptimal neck coverage due to inability to conform the crossed devices to the individual parent vessel anatomy and junctional geometry, ultimately resulting in high thromboembolic risk and unsatisfactory embolization (6–9). Given these serious disadvantages, other alternative technical solutions for endovascular treatment of WNA have emerged in the recent years. The main goal of the novel techniques is to reduce complexity of the procedure, improve obliteration rates, and reduce the amount of metal in the parent vessel. One such option is the Barrel VRD—a laser cut self-expanding nitinol stent. The device has a bulged middle section that protrudes into the aneurysmal neck, providing a single-device neck protection for coil embolization. The stent is mounted on a 0.016-inch nitinol pusher wire and is delivered through a 0.021-inch microcatheter. The Barrel VRD is completely retrievable up to 3 times over its full length and has a double spiral strut that helps navigation in tortuous anatomy. We didn't experience any technical difficulties in delivering the Barrel VRD device.

The safety and efficacy of the Barrel VRD has been demonstrated in prior studies. A 2016 case series of 17 patients with 17 aneurysms by Mühl-Benninghaus et al. showed complete occlusion rate of 64.8% and residual neck of 29.4% (10). In another case series of 19 patients with 19 aneurysms, the adequate occlusion rate after 12 months follow-up was assessed to be 78.9% and the neurological complications to 5% (11). Another recent multi-center study showed even better results (12). The authors presented 21 cases with 21 aneurysms with 90% immediate complete occlusion rate and 95% complete occlusion rate after a median follow up of 282 days follow up.

Other devices designed to provide optimal neck coverage for coil-embolization of WNA include the PulseRider (Pulsar Vascular, USA), the pCONus 1 and 2 (Phenox Germany), the pCANvas (Phenox, Germany), and the eCLIPs device (Evasc, Canada). The pCONus is reported as safe with low recanalization rate at short to midterm follow-up of 86.3–96.7% (13, 14). On the other hand, this device as well as the “waffle cone technique,” has the theoretical disadvantage to direct blood flow into the aneurysmal lumen, potentially increasing the risk of recanalization. The pCANvas was developed as an answer to this question, as it has a square membrane on its distal end, designed to further promote aneurysm thrombosis by limiting the intra-saccular flow.

The eCLIPs device combines flow diversion effect with neck bridging properties, but it appears to have a greater learning curve with 76% technical success rate and 73,9% adequate occlusion rate on midterm follow up (15). The ANSWER (Adjunctive Neurovascular Support of Wide-necked aneurysm Embolization and Reconstruction) paper demonstrated similar midterm results after treatment with the PulseRider device (16).

Aside from neck-bridging devices, intra-aneurysmal blood flow disrupting device can be considered for treatment of WNA. One such example is the WEB device—a highly innovative flow disrupter, with reported adequate occlusion at the 1 year mark of 80–82% and periprocedural morbidity of 1.8–2.7% (17, 18). However, the WEB device is not designed for treatment of previously coiled recanalized aneurysms.

In our case, we selected Barrel VRD among all available neck-birding devices and techniques mainly due to its unique design and ease of delivery. We predicted that the bulged portion of the stent would adopt to the concave shape of the recanalized aneurysm and it would provide ideal neck coverage and solid support by protruding into the large coil mass. This particular feature was considered superior to all other devices in terms of prevention of coil herniation. In addition, the junctional angle between the A1/A2 was favorable for optimal positioning of the distal and proximal ends of the device in the parent vessels. As described above, the device placement and the angiographic and clinical results of the procedure were very satisfactory, ultimately resulting in complete and permanent obliteration of the aneurysm.

## Conclusion

The Barrel Vascular Reconstruction Device (VRD) may be particularly useful for treatment of previously embolized and recanalized wide-neck aneurysm, harboring large intrasaccular coil mass. Given its ease of deployment and satisfactory immediate and midterm results, the Barrel VRD can be a good alternative to other traditionally used neck-bridging techniques and devices.

## Author Contributions

SS drafted the original manuscript version, reviewed all suggestions provided by all co-authors, approved the final version, and agreed to be accountable for all aspects of the work in ensuring that questions related to the accuracy or integrity of any part of the work are appropriately investigated and resolved. RR provided major edits and assumed responsibility for final review and submission as a corresponding author. All co-authors provided substantial contribution to the manuscript as physicians who were directly involved in the care of this patient, contributed with revisions to the original draft, approved the final version of the manuscript, and agreed to be accountable for all aspects of the work.

### Conflict of Interest

The authors declare that the research was conducted in the absence of any commercial or financial relationships that could be construed as a potential conflict of interest.
